# www.common-metrics.org: a web application to estimate scores from different patient-reported outcome measures on a common scale

**DOI:** 10.1186/s12874-016-0241-0

**Published:** 2016-10-19

**Authors:** H. Felix Fischer, Matthias Rose

**Affiliations:** 1 Department of Psychosomatic Medicine, Clinic for Internal Medicine, Charité Universitätsmedizin Berlin, Berlin, Germany; 2 Institute for Social Medicine, Epidemology and Health Economics, Charité Universitätsmedizin Berlin, Berlin, Germany; 3 Department of Quantitative Health Sciences, University of Massachusetts Medical School, Worcester, USA

**Keywords:** Item-Response Theory, Measurement, Patient Reported Outcomes, Depression, Anxiety, Physical function

## Abstract

**Background:**

Recently, a growing number of Item-Response Theory (IRT) models has been published, which allow estimation of a common latent variable from data derived by different Patient Reported Outcomes (PROs). When using data from different PROs, direct estimation of the latent variable has some advantages over the use of sum score conversion tables. It requires substantial proficiency in the field of psychometrics to fit such models using contemporary IRT software. We developed a web application (http://www.common-metrics.org), which allows estimation of latent variable scores more easily using IRT models calibrating different measures on instrument independent scales.

**Results:**

Currently, the application allows estimation using six different IRT models for Depression, Anxiety, and Physical Function. Based on published item parameters, users of the application can directly estimate latent trait estimates using expected a posteriori (EAP) for sum scores as well as for specific response patterns, Bayes modal (MAP), Weighted likelihood estimation (WLE) and Maximum likelihood (ML) methods and under three different prior distributions. The obtained estimates can be downloaded and analyzed using standard statistical software.

**Conclusions:**

This application enhances the usability of IRT modeling for researchers by allowing comparison of the latent trait estimates over different PROs, such as the Patient Health Questionnaire Depression (PHQ-9) and Anxiety (GAD-7) scales, the Center of Epidemiologic Studies Depression Scale (CES-D), the Beck Depression Inventory (BDI), PROMIS Anxiety and Depression Short Forms and others. Advantages of this approach include comparability of data derived with different measures and tolerance against missing values. The validity of the underlying models needs to be investigated in the future.

## Background

One of the major developments in the recent years of Patient-Reported Outcome (PRO) measurement has been the adoption of methods based on Item-Response Theory (IRT) [1]. Those methods have been used to develop shorter measures [2], to apply computer-adaptive tests [3] or to assess systematic differences in response behavior between groups [4]. One of the core advantages of IRT compared to Classical Test Theory (CTT) is the possibility to estimate common models for different PROs measuring the same constructs, allowing comparisons of the measured construct over different measures [1]. We call IRT models that comprise the item parameters from items of various measures, measuring a common variable, “common metrics”. With such statistical models, one can estimate the variable of interest by subsets of items, e.g. when different measures are used or when data is missing.

In the recent years such models have been developed in various domains: physical functioning [5–7], pain [8, 9], fatigue [10], headache [11], anxiety [12] and depression [13–16]. A promising field of research is the linking of pediatric and adult measures to allow meaningful comparisons over the course of time [17]. Different methods yielding comparable results have been applied to link measures, such as fixed-parameter estimation or concurrent estimation with subsequent linking [12, 13, 18]. So far, those IRT models have been frequently used to develop sum score conversion tables between measures [7, 8, 10, 12, 15] since it is possible to derive latent trait estimates solely from the sum score [19]. It is also possible to estimate the latent trait directly from the response pattern. This approach has some advantages over the use of sum score conversion tables since it takes into account differences in the response pattern, yielding more accurate results [12, 13] than converted sum scores. It also is favorable in case of missing item response, since estimation of the latent variable is still viable under that condition [12, 13].

Estimation of IRT scores based on common metrics can currently be done in a number of different statistical packages, such as IRTPRO, PARSCALE, R or SAS. Nonetheless, it requires substantial proficiency in the field of psychometrics to fit those models, hampering accessibility of common metrics for researchers from other fields. We developed a web application (http://www.common-metrics.org), which allows estimation of latent variable scores more easily using such common metrics.

Our goal is to enable researchers to compare data obtained with different measures, for example if in Study A the Patient Health Questionnaire 9 (PHQ-9) has been used for the measurement of depression, but in Study B the Beck Depression Inventory (BDI) was the measure of choice. In this paper, we describe the general organization of the application, the technical details of the implemented estimation as well as aspects of data safety. Finally, advantages and caveats of the application are discussed.

## Implementation

### Overview

**Fig. 1:**
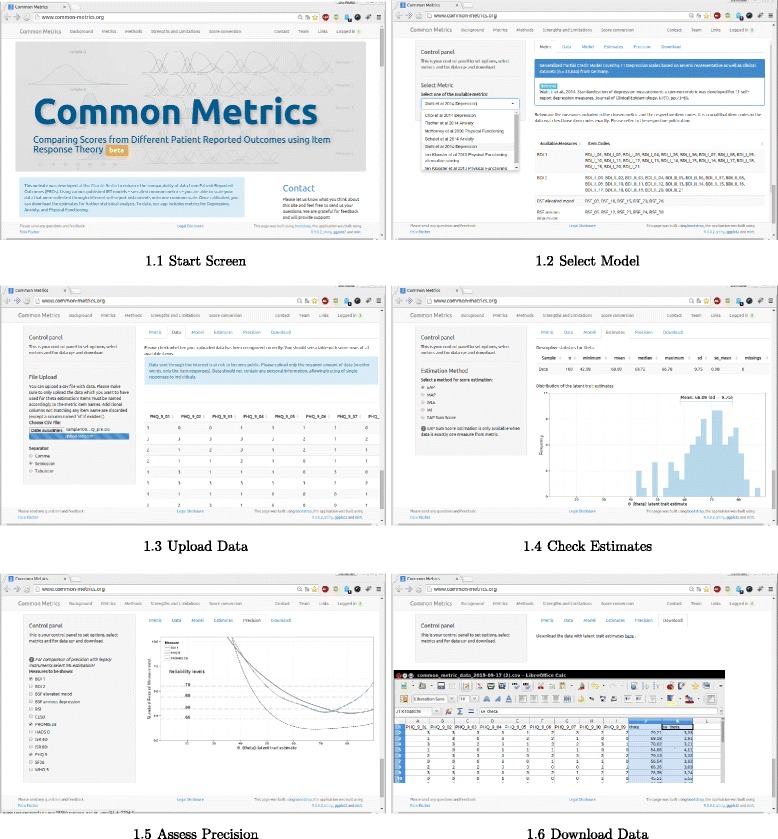
Overview over the application workflow

The application itself consists of a control panel and 6 tabs (see Fig. 1).

- Metric: select one of the available metrics and review the item codes for each measure. Currently, we implemented common metrics for the measurement of depression [13, 14], anxiety [12, 20], and physical functioning [5, 7] containing measures such as the Patient Health Questionnaire Depression (PHQ-9) and Anxiety (GAD-7) scales [21, 22], the Center of Epidemiologic Studies Depression Scale (CES-D) [23], the Beck Depression Inventory (BDI) [24], PROMIS Anxiety and Depression Short Forms [25–27] and others. We provide some information about those metrics, such as estimation sample size and included items, but users are referred to the actual publications. Additional metrics can be added if requested.
- Data: select example data or upload your own dataset. The identification of items in the dataset is case-sensitive and column names must match the item codes exactly. Each row corresponds to one observation.
- Model: select prior distribution (N(0,1), N(0,10) and estimated from data) and review item parameters.
- Estimates: select estimation method EAP (expected a posteriori), MAP (Bayes modal), WLE (Weighted likelihood estimation), ML (Maximum likelihood) or EAP Sum Score) and review descriptive statistics (n, min, mean, median, maximum, standard deviation, standard error of the mean, percentage of missing values) including a histogram of the distribution of latent trait estimates.
- Precision: review precision of estimates (standard error) over latent variable continuum. If estimation method is maximum likelihood (ML), test precision of legacy instruments can be shown.
- Download: download dataset with score estimate and standard error of measurement.

The default estimator selection (EAP with N(0,1) prior) can be considered as current standard and is appropriate for a wide range of applications. However, we allow the selection of different estimators and priors, since those might be more appropriate in a given situation. For example, comparison of the precision of a set of items to legacy instruments is only meaningful under ML estimation. Since the application is solely intended to allow researchers to estimate latent trait scores on several previously published common metrics, the application does not include any possibility to reestimate the underlying item parameters.

### Technical details of theta estimation

The application sets up the respective IRT model (Graded Response Model or Generalized Partial Credit Model) with all parameters fixed to the item parameters of the desired common metric. Prior distribution can be selected by the user. The underlying R package mirt [28] uses a marginal maximum likelihood method to estimate item parameters of IRT models, hence, estimation of person parameters can be conducted independently. For person parameter estimation we included the sum score as well as response pattern expected a posteriori (EAP), Bayes modal (MAP), Weighted likelihood estimation (WLE) and Maximum likelihood (ML) methods. Theta estimates and standard errors are transformed to the t-metric (mean 50, standard deviation of 10). For some metrics, 50 is some meaningful anchor point like the general population mean [12–14]. Test specific standard errors were calculated for models comprising all items from one questionnaire. Please note that these standard errors are valid under ML estimation only.

The website was build using R 3.0.2 [29], Shiny [30] and ggplot2 [31]. IRT models used for theta estimation were estimated using the R-package mirt [28].

### Data safety

From uploaded data, all columns are disregarded if their name does not match any of the item codes available in the selected metric. Although we do not save uploaded data beyond the need for processing within the actual session, users must be aware that sensible data sent through the internet is a potential security risk and data might become public. We hence advise user to upload only the required amount of data (in other words, only the item responses) and ensure that uploaded data fulfills data safety standards. Data should not contain any personal information, allowing tracing of single responses to individuals.

The application was approved in its current version by the data protection commissioner of the Charité Universitätsmedizin Berlin, Germany.

## Results

We present a website that allows the use of common metrics to estimate latent variable on a common scale independently from the measure being used. Compared to traditional IRT software the major strength of our approach by providing a web application is that theta estimation from different PROs does not require detailed knowledge on IRT modeling nor estimation techniques. We provide a simple interface to check basic summary data and data may later be used in any other software the user is familiar with, such as Excel, SPSS, SAS or R.

The approach implemented in www.common-metrics.org in general promises a number of advantages compared to the use of instrument dependent sum scores, such as

1. comparability of data derived with different measures, e.g. when assessing routine data or in case of meta-analysis on primary data level
2. more precise measurement (i.e. decreased standard error of individual estimate) by taking the response pattern into account as well as when using two or more measures
3. tolerance against missing values
4. increased validity of the scale compared to instrument dependent scales.

However, users should be aware of the limitations of this approach. One issue is the validity of the underlying model. Although findings like the overlap of different cut-off values from static measures on the common metric make us confident in the validity of some of the models [12–14], a general lack of external validation studies must be acknowledged. However, providing a technical basis to use such models in research more easily might be a catalyst for such validation studies.

Furthermore, one must be aware that measures differ in their coverage over the theta continuum. While it has been shown that the use of IRT estimates instead of sum scores leads to similar results [1, 20], use of different measures instead of the same to estimate theta showed in one study a notable impact on the effect estimate [32]. This can lead to severe bias when comparing scores from tests with differing precision over the continuum. Since most instruments were developed in clinical samples this might be especially problematic in relatively healthy samples, such as the general population. A possible solution is to take the uncertainty about the theta estimate – its standard error – into account, e.g. in a Bayesian framework or adopting the plausible value approach [33–35]. This issue must be investigated in the near future.

Another thread to validity is the possibility of differential item functioning between the samples which were used for model calibration and the samples used in application. For example, it is unclear whether common metric developed from German samples [14] can be used in English speaking samples as well. However, this problem is also apparent in the use of sum score conversion tables.

## Conclusion

We firmly believe that common metrics including a variety of measures have a much stronger chance to become valid and accepted standards for a specific domain rather than a single questionnaire. We hope this website shows the potential that the development of common metrics holds, facilitates studies investigating the validity and clinical usefulness of such metrics and contributes to the movement towards instrument independent scales in measurement of Patient-Reported Outcomes.

## Availability and requirement

Our web application is available at http://www.common-metrics.org with information about the background, methods, and limitations of this approach. The application may be freely used to estimate theta scores on a common metric.
